# Gaseous- and Condensed-Phase Activities of Some Reactive P- and N-Containing Fire Retardants in Polystyrenes

**DOI:** 10.3390/molecules28010278

**Published:** 2022-12-29

**Authors:** Svetlana Tretsiakova-McNally, Aloshy Baby, Paul Joseph, Doris Pospiech, Eileen Schierz, Albena Lederer, Malavika Arun, Gaëlle Fontaine

**Affiliations:** 1 Belfast School of Architecture and the Built Environment, Ulster University, 2-24 York Street, Belfast BT15 1AP, Northern Ireland, UK; 2 Institute for Sustainable Industries and Liveable Cities, College of Engineering and Science, Werribee West Campus, Victoria University, P.O. Box 14428, Melbourne, VIC 8001, Australia; 3 Leibniz-Institut für Polymerforschung Dresden, Hohe Straβe 6, 01069 Dresden, Germany; 4 Department of Chemistry and Polymer Science, Stellenbosch University, Matieland 7602, South Africa; 5 CNRS, INRAE, Centrale Lille, UMR 8207-UMET-Unité Matériaux et Transformations, University of Lille, F-59000 Lille, France

**Keywords:** polystyrene, thermal decomposition, reactive modification, P- and N-containing fire retardants, mode of action of fire retardance, gaseous-phase inhibition, char formation

## Abstract

Polystyrene (PS) was modified by covalently binding P-, P-N- and/or N- containing fire-retardant moieties through co- or ter-polymerization reactions of styrene with diethyl(acryloyloxymethyl)phosphonate (DEAMP), diethyl-*p*-vinylbenzyl phosphonate (DEpVBP), acrylic acid-2-[(diethoxyphosphoryl)methylamino]ethyl ester (ADEPMAE) and maleimide (MI). In the present study, the condensed-phase and the gaseous-phase activities of the abovementioned fire retardants within the prepared co- and ter-polymers were evaluated for the first time. Pyrolysis–Gas Chromatography/Mass Spectrometry was employed to identify the volatile products formed during the thermal decomposition of the modified polymers. Benzaldehyde, α-methylstyrene, acetophenone, triethyl phosphate and styrene (monomer, dimer and trimer) were detected in the gaseous phase following the thermal cracking of fire-retardant groups and through main chain scissions. In the case of PS modified with ADEPMAE, the evolution of pyrolysis gases was suppressed by possible inhibitory actions of triethyl phosphate in the gaseous phase. The reactive modification of PS by simultaneously incorporating P- (DEAMP or DEpVBP) and N- (MI) monomeric units, in the chains of ter-polymers, resulted in a predominantly condensed-phase mode of action owing to synergistic P and N interactions. The solid-state ^31^P NMR spectroscopy, Scanning Electron Microscopy/Energy Dispersive Spectroscopy, Inductively-Coupled Plasma/Optical Emission Spectroscopy and X-ray Photoelectron Spectroscopy of char residues, obtained from ter-polymers, confirmed the retention of the phosphorus species in their structures.

## 1. Introduction

Polystyrene, PS, is the main component of the foamed materials that are widely used in the built environment for thermal insulation purposes. The main drawback of using PS is its relatively high levels of the ease of ignition and flammability, often leading to an extremely fast fire spread in real-life fire situations. Combustion of PS generally results in the large volumes of toxic smoke and produces mixtures of gases containing styrene-monomer and other compounds of low molecular weights, which can also undergo flaming combustion [1,2]. In addition, while burning, PS can melt-flow and drip, thus creating a secondary fire hazard and can thus result in the propagation of the flames [3]. Fire Safe Europe has recently highlighted that it takes just about three minutes for a small fire in a building to get to a flashover [4]. Therefore, it is of a paramount importance to modify the commercial polymers (including PS) used as construction elements, with a suitable fire retardant (FR) in order to slow down the thermal decomposition and to restrict the evolution of large amounts of flammable volatiles. This could be achieved either by physically mixing the required amounts of a FR with a polymer matrix, or by chemically binding the FR moieties onto the main chains of PS [5,6]. The latter methodology includes co-polymerization of styrene with those unsaturated compounds that can impart higher thermal stability to the parent polymer. Over the last two decades, polymer industry relied heavily on halogenated additive FRs [2,6,7]. However, the use of this class of FRs is restricted, or even prohibited, due to the concerns related to environmental toxicity and bioaccumulation [6,8,9]. The alternative, non-halogenated formulations have been developed by many research groups to resolve some of these issues [5,6,10,11,12]. 

Over the years, we have been focusing our research efforts on P- or P-N- containing polymerizable compounds, such as organic phosphonates, phosphorylamino esters and phosphates [8,11,13,14,15,16,17]. As reported previously, the thermal stability and fire retardance of polyacrylonitrile, poly(methyl methacrylate) and polystyrene improved as a result of co-polymerization reactions of P-based FRs with the corresponding monomers [13,14,15,16,17]. For example, as reported in [13], the reactive modification of PS, through co-polymerization with phosphorylamino ester (where P and N atoms are bonded within the same FR group), reduced its flammability by almost 25%, at a very low level of P loading (0.2 wt. %) in the polymer [14]. However, to the best of our knowledge, there were no studies on ter-polymerization processes, when P and N atoms, within different monomeric units, can be incorporated simultaneously into the polymeric chains of PS. Thus, for the first time, we have carried out not only co-polymerization but also ter-polymerization of styrene with nominal quantities of diethyl(acryloyloxymethyl) phosphonate (DEAMP), diethyl-*p*-vinylbenzylphosphonate (DEpVBP) and maleimide (MI) [8]. The impact of such modifications on the thermal decomposition and combustion of PS was reported earlier [8]. It was found that the thermal stability and combustion characteristics of PS were significantly altered by the presence of the nominal amounts of P- and N- containing units in the polymeric chains. For instance, at 800 °C, in a nitrogen atmosphere, the char formation by ter-polymers, as revealed through thermogravimetric analysis (TGA), increased 44 times, from 0.5 wt. % for PS up to 22 wt. % for ter-polymers. In addition, the heat release rates, total heat releases and heat release capacities of the ter-polymers, determined using the pyrolysis combustion flow calorimetry, were reduced by almost 50% in comparison to the same parameters measured for the unmodified PS [8]. 

Evaluation of the possible mechanisms of fire retardance is essential for the development of new effective formulations for passive fire protection of the polymers. It is known that the condensed-phase activity of phosphorus FRs can involve charring, facilitated by the dehydration of the polymeric structure, leading to aromatization, aromatic ring fusion, cross-linking and graphitization [18,19]. The cross-linking of polymeric chains can also be triggered by the by-products formed during the decomposition of phosphorus FRs [11]. In the case of polyolefins, the P-based compounds tend to act mainly in the gaseous phase, by scavenging active H⋅ and HO⋅ radicals and preventing their oxidation. The deciphering of the condensed- and gaseous- phase activities of FRs is a very challenging task as their decomposition may produce new, reactive intermediates that consequently may recombine into the unexpected by-products [20].

**Figure 1:**
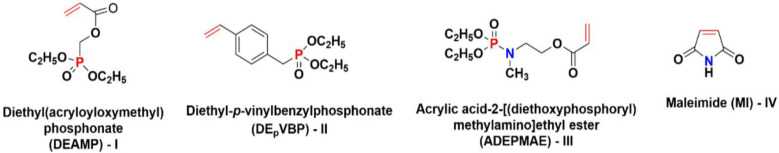
The chemical structures of P- containing (**I**–**III**) and N-containing (**VI**) monomers.

The present study was aimed at gaining a better understanding of the decomposition pathways of polystyrenes reactively modified through co- and ter-polymerization of styrene with nominal amounts (10 mol. % in the feed) of DEAMP, DEpVBP, acrylic acid-2-[(diethoxyphosphoryl)methylamino]ethyl ester (ADEPMAE) and/or MI (Figure 1). Apparently, there are no reports in the literature dealing with the mechanisms of action of selected P- and N-containing FRs within the styrenic polymers. The analytical investigations carried out in the current research included: identification of pyrolysis gases and decomposition fragments with the aid of Pyrolysis–Gas Chromatography/Mass Spectrometry (Py-GC/MS) and char analyses using solid-state ^31^P Nuclear Magnetic Resonance (NMR) spectroscopy, Scanning Electron Microscopy/Energy Dispersive Spectroscopy (SEM/EDS), Inductively-Coupled Plasma/Optical Emission Spectroscopy (ICP/OES) and X-ray Photoelectron Spectroscopy (XPS).

## 2. Results and Discussion

It is established that, generally, the heating of the unmodified polystyrene (PS) results in a random scission of the main chains. Figures S1 and S2 (Supplementary Information, SI) show the gas chromatograms and the mass spectrum of the gaseous products formed upon the decomposition of PS at 260 °C and 415 °C. The temperature points were selected based on the data of thermogravimetric analysis (TGA) at 10 °C/min [8]. The proposed assignment of the retention times (RT) and mass peaks (*m*/*z*) obtained through Pyrolysis-Gas Chromatography/Mass Spectrometry (Py-GC/MS) of PS is given Table 1; these correlate well with the published data [7,21,22,23]. As follows from the results given in Table 1, Figures S1 and S2, the major pyrolysis gases were identified as styrene-monomer, styrene-dimer and styrene-trimer as well as benzaldehyde, acetophenone and α-methylstyrene. The formation of these volatiles was also previously reported by us while investigating the thermal decomposition of polystyrenes [11,15,16]. The other detected volatiles and fragments included: benzene, toluene, ethylbenzene, limonene, stilbene, bibenzyl, and diphenylpropane and diphenylpropene. The minor components originating from the solvent or initiator residues were also identified but not listed in Table 1. 

**Figure 2:**
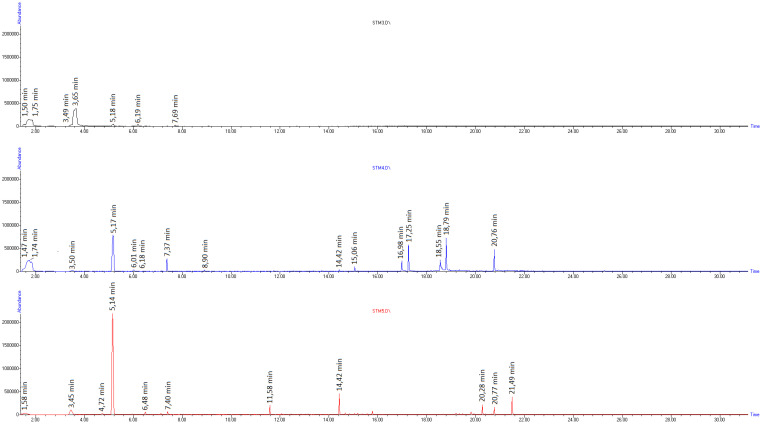
Gas chromatograms of pyrolysis gases released upon the decomposition of poly(S-*co*-DEAMP) at 210 °C (black), 325 °C (blue) and 380 °C (red).

**Figure 3:**
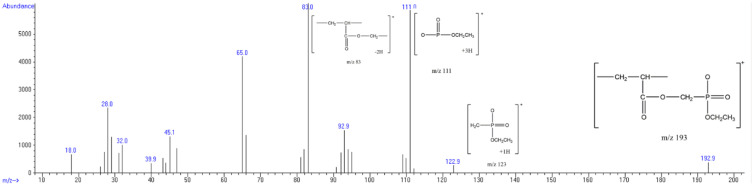
Mass spectrum of gases formed as a result of poly(S-*co*-DEAMP) pyrolysis at 325 °C (6.006 min).

In the case of the modified polymer, poly(S-*co*-DEAMP), the increase of temperature from 210 °C to 325 °C and then to 380 °C, resulted in the relative enhancement of chromatographic peaks corresponding to styrene-monomer. Nevertheless, the peaks corresponding to styrene-dimer and styrene-trimer, visible on the chromatogram at 325 °C, seemed to be drastically reduced at 380 °C (Figure 2). The GC peaks corresponding to toluene, benzaldehyde and acetophenone were also significantly reduced, or almost completely disappeared, at 325 °C and 380 °C. Compared to the chromatographic pattern of the unmodified PS, some new peaks were found for this co-polymer including ethanol (RT 1.75 min, *m*/*z* = 46); compounds with fused aromatic rings, i.e., indene (RT 7.40 min, *m/z*=116) and α-tetralone (RT 11.58 min, *m*/*z* = 146); higher carboxylic acids or their esters (RTs in the range from 16.98 to 18.79 min). Additionally, a P-containing fragment with RT of 6.01 min and *m/z* = 193 (Figure 3) was detected at 325 °C. The proposed structures of other fragments, which can arise from pendent DEAMP groups of the co-polymer, are shown beside the corresponding *m*/*z* peaks in Figure 3. It is likely that the fragment with *m/z* = 28, absent in the mass spectrum of the unmodified PS, was produced *via* a process of *cis*-elimination associated with a loss of ethylene molecules from ethyl groups of DEAMP, as discussed earlier [15]. There was also an abundance of a fragment (*m*/*z* = 65), possibly corresponding to the PO_2_H_2_ intermediate formed by the recombination of PO⋅ (*m*/*z* = 47), HO⋅ and H⋅ radicals [20]. Thus, the presence of the phosphonate groups, from DEAMP, in the polymer structure appeared to be markedly reduced the production of major volatiles at elevated temperatures.

**Figure 4:**
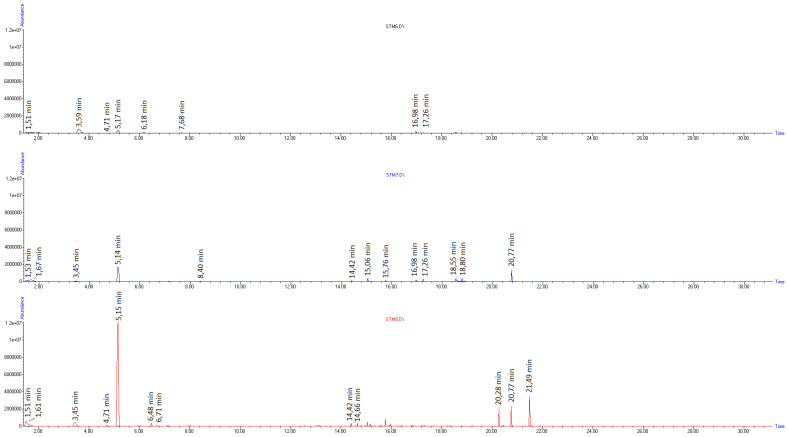
Gas chromatograms of pyrolysis gases released upon the decomposition of poly(S-*co*-DEpVBP) at 240 °C (black), 355 °C (blue) and 445 °C (red).

The modification of PS chains with benzylic phosphonate (DEpVBP) moieties also resulted in the reduced number of chromatographic peaks associated with the main gaseous products from the decomposition of the co-polymer (Figure 4). As temperature increased from 250 °C to 355 °C and to 445 °C, styrene-monomer production was found to be enhanced, and concomitantly the dimeric species were registered at 355 °C (Figure S3, SI), which subsequently disappeared at 445 °C (Figure S4, SI). However, the production of styrene-trimer and toluene seemed to be on the increase, whilst benzaldehyde and acetophenone peaks disappeared as the temperature was raised. For this co-polymer, the pyrolysis gases and molecular ions were very similar to those identified for poly(S-*co*-DEAMP) except for a new GC peak that can be assigned to triethyl phosphate (TEP), as evident at 355 °C in the gaseous phase (RT 8.40 min, *m*/*z* = 182) (Figure 4 and Figure S3 in SI). It is worthwhile noting that for poly(S-*co*-DEpVBP) the fragments associated with benzylic phosphonate monomeric units (*m*/*z* = 254) were observed in the relevant mass spectra. A similar trend was found for poly(S-*co*-DEAMP), when monomeric DEAMP fragments (*m*/*z* = 222) were detected among the pyrolysis gases.

**Figure 5:**
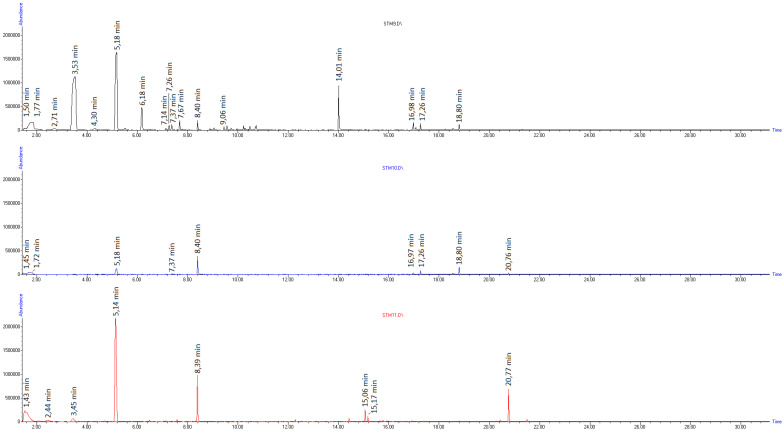
Gas chromatograms of pyrolysis gases released upon the decomposition of poly(S-*co*-ADEPMAE) at 250 °C (black), 300 °C (blue) and 400 °C (red).

Apparently, the incorporation of ADEPMAE units within the polymeric chains of PS lowered styrene-monomer production, as seen from the chromatograms recorded at 250 °C and 300 °C (Figure 5). In addition to this, the dimer and trimer production was observed only at 400 °C, whilst the peaks corresponding to toluene, benzaldehyde and acetophenone appeared to be reduced to near-zero upon heating. Interestingly, here, TEP (RT 8.39–8.40 min, *m/z* = 182) was detected in the gaseous phase at all three temperatures following the thermal decomposition of poly(S-*co*-ADEPMAE). The production of TEP is likely to be responsible for the enhanced gaseous-phase fire-retardant activity. Overall, for this co-polymer, the chromatographic patterns revealed a further reduction of pyrolysis gases, which, possibly, can be explained by considering the cooperative effects of P and N atoms of the P-N bond within ADEPMAE groups.

**Figure 6:**
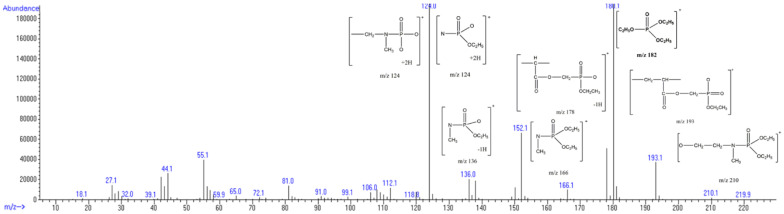
Mass spectrum of gases formed as a result of poly(S-*co*-ADEPMAE) pyrolysis at 250 °C (14.003 min).

The proposed structures of some of the fragments associated with the breakdown of ADEPMAE modifying group are given in the mass spectrum recorded at 250 °C, the temperature point at which this group begins to act as a fire retardant (Figure 6 and Table 2).

**Scheme 1:**
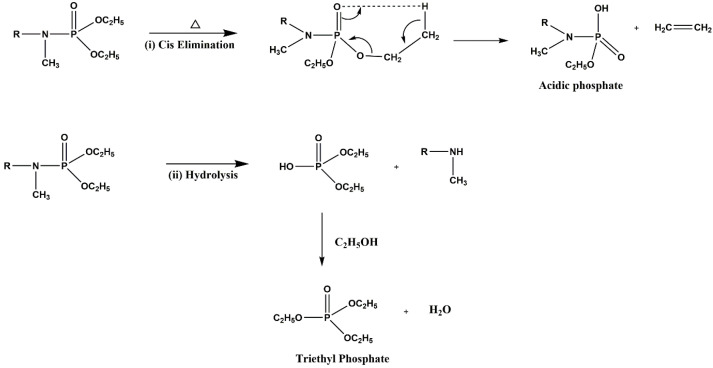
Proposed decomposition routes of ADEPMAE moeities in the gaseous phase.

Here, we propose two routes for the thermal decomposition of the co-polymer containing ADEPMAE. The first route considers the phosphonate side groups undergoing a reaction of *cis*-elimination, *via* a five-membered cyclic intermediate, and rearrangement to form acidic phosphate and ethylene (Scheme 1(i)). The production of ethylene and acidic species was confirmed by MS detecting the corresponding molecular ions (Figure 6 and Table 2). In the second route (Scheme 1(ii)), the presence of protic species can, potentially, promote hydrolysis of the P-N bonds under acidic conditions [24]. Diethyl phosphate formed after the hydrolysis can then react with ethanol (confirmed by MS) and form TEP (Scheme 1(ii)).

The chromatographic patterns (Figure S5, SI) of the co-polymer with built-in MI groups, poly(S-*co*-MI), were more or less similar to those obtained for the unmodified PS, indicating that the incorporation of MI did not drastically impact the pyrolysis process. However, several N-containing fragments were possibly produced as shown in Table 2 and in the mass spectrum (Figure S6, SI).

When both phosphorus (DEAMP) and nitrogen (MI) units were chemically bonded to the chains of PS, within the ter-polymer poly(S-*ter*-DEAMP-*ter*-MI), there was no evidence of any new GC peaks (Figure S7, SI) apart from those already detected in the homopolymer and the corresponding co-polymers. As it follows from Figure S8 in SI, the signals assigned to the P-containing intermediates with *m*/*z* = 178 and *m*/*z* = 193 (Table 2) were observed, similar to the fragments found for the poly(S-*co*-DEAMP). Most importantly, for this ter-polymer, the evolution of all the major and minor volatiles was drastically reduced, with some components not detected at all in the gaseous phase, as temperature increased from 240 °C to 320 °C to 375 °C (Figure S7). 

In the case of the ter-polymer containing benzylic monomeric units DEpVBP and MI, the number of chromatographic peaks was also significantly reduced (Figures S9 and S10, SI). However, the main volatiles identified for PS were still present at 270 °C, and styrene-dimer was present at both 270 °C and 375 °C. Similar to the corresponding co-polymer, poly(S-*co*-DEpVBP), the formation of TEP responsible for the inhibitory actions was detected. In addition, the Py-GC/MS runs did not register any P-N-containing fragments, or other new fragments, among the evolved gas species. This could imply that the enhanced charring, initially detected through TGA [8], was blocking the release of the volatiles to the gaseous phase, for both ter-polymers. The abovementioned results, as well as the thermal and combustion characteristics of the studied ter-polymers [8] may suggest that the condensed-phase effect is significantly prevailing over the gaseous-phase mode of action. 

A better understanding of the possible mechanistic pathways can be gained by employing analytical techniques to characterize the char residues obtained upon the thermal decomposition of the modified polymers, such as poly(S-*co*-ADEPMAE), poly(S-*ter*-DEAMP-*ter*-MI), and poly(S-*ter*-DEpVBP-*ter*-MI). The retention of phosphorus within the residual chars usually points towards a condensed-phase activity of the modifying groups. The use of the solid-state ^31^P NMR can thus assist in deciphering transformations of the fire-retardant groups. The corresponding NMR spectra of the chars are given in Figure S11, SI. The signal with a chemical shift δ of 0 ppm in the ^31^P NMR spectra of chars formed by the poly(S-*co*-ADEPMAE) and ter-polymers, poly(S-*ter*-DEAMP-*ter*-MI) and poly(S-*ter*-DEpVBP-*ter*-MI), can be assigned to the ‘phosphorus’ acid generated during the thermal cracking of the phosphonate groups [11,15,25,26,27,28]. Generally, the peak at δ = 0 ppm can be assigned to the unbound phosphoric acid [15,27]. The ^31^P signals were shifted to the negative values, δ = −10 ppm, in the NMR spectra of chars obtained from the co-polymer with ADEPMAE (Figure S11a) and from the ter-polymer, poly(S-*ter*-DEpVBP-*ter*-MI) (Figure S11c). This could be explained by a possible condensation of ‘phosphorus’ acidic moieties, producing oligomeric or polymeric species. The generated P-acids may phosphorylate aromatic rings of the modified polystyrenes, subsequently forming char precursors and cross-linking polymeric chains [29]. It is highly relevant to note that the char from poly(S-*ter*-DEAMP-*ter*-MI) did not show the presence of oligo- or poly- phosphoric acids in the condensed phase, implying, possibly, a different mode of action of DEAMP units when present along with the MI ones. The signal, or a shoulder of the main signal, recorded at δ 19.1 ppm and 21.3 ppm (Figure S11b,c) could possibly arise from the non-cracked (undecomposed) phosphonate units of DEAMP and DEpVBP, respectively [28]. This was confirmed by comparing these chemical shifts with the corresponding signals, previously observed in the ^31^P NMR solution-state spectra, which were δ = 19.0 ppm for DEAMP and δ = 26.1 ppm for DEpVBP [8,15,16,17]. Thus, the NMR spectroscopy confirmed the retention of phosphorus in the char residues obtained from ter-polymers and from the co-polymer bearing the P-N bond in the modifying monomeric unit.

**Figure 7:**
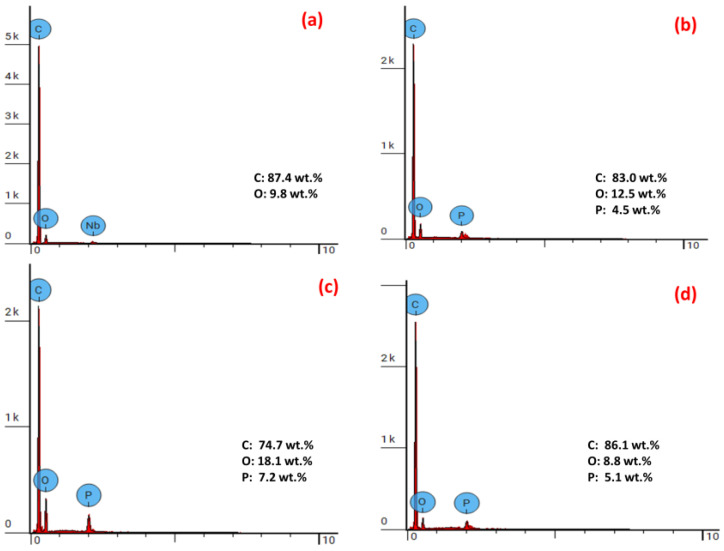
The EDS spectra of char residues obtained from the samples of: PS (**a**), poly(S-*co*-ADEPMAE) (**b**), poly(S-*ter*-DEAMP-*ter*-MI) (**c**), and poly(S-*ter*-DEpVBP-*ter*-MI) (**d**). All the samples were sputtered with gold (Au).

The formation of a resilient char could effectively reduce the heating of the underlying polymer matrix and prevent gases from entering the pyrolysis zone, thereby, possibly, providing a retardant effect *via* condensed-phase actions [30,31,32]. The EDS data for the chars produced by the modified polystyrenes showed that P content varied from 4.5 to 7.2 wt. % (Figure 7). This indicate that phosphorus species formed during thermal decomposition were retained in char residues, which was also evident from the solid-state ^31^P NMR. As suggested previously, the modifying groups may form phosphoric, or polyphosphoric, acids and other phosphorus compounds at higher temperatures, thus remaining in chars after the thermal decomposition. As a result, the char surfaces became enriched with phosphorus, providing heat insulation and promoting catalytic reactions to enhance the fire resistance of PS.

The increased phosphorus contents in the residual chars obtained from the modified polymers were also confirmed by the ICP-OES technique. The average values of char yields, P contents in chars and the percentage of actual P retentions are summarized in Table 3. The P contents in the studied polymers, calculated from solution-state ^1^H NMR spectral data [8], are also provided in Table 3. The actual retention of P in chars could indicate the extent of the condensed-phase activity of the modifying groups. The results clearly demonstrate that the char residues contained higher amounts of phosphorus compared to the values found in the corresponding co- and ter-polymers, prior to the thermal decomposition. This indicate that the phosphorus species mainly retained in the structures of chars, which was also evident from the solid-state ^31^P NMR spectra and EDS. The highest actual retention of P was registered in the char formed by poly(S-*co*-DEAMP). In the case of chars obtained from poly(S-*co*-ADEPMAE), the P retention in the condensed phase was the lowest among the studied modified polystyrenes, which, possibly, suggests the prevailing role of the gaseous-phase mode of action. This correlates with Py-GC/MS results of poly(S-*co*-ADEPMAE) that indicated the inhibitory activity of TEP in the gaseous phase.

The X-ray photoelectron spectroscopy (XPS) is a valuable tool to characterize the charred polymeric substrates in terms of elemental composition and chemistry. In the present study, the XPS was employed to gain a deeper insight into the elemental compositions and natures of char residues from ter-polymers, poly(S-*ter*-DEAMP-*ter*-MI) and poly(S-*ter*-DEpVBP-*ter*-MI). The full width at half maximum (FWHM) parameter was maintained between 1.2–1.8 during the interpretation of the spectra. Table 4 provides the detailed data regarding the carbon and oxygen bonds present in the chars of ter-polymers.

**Figure 8:**
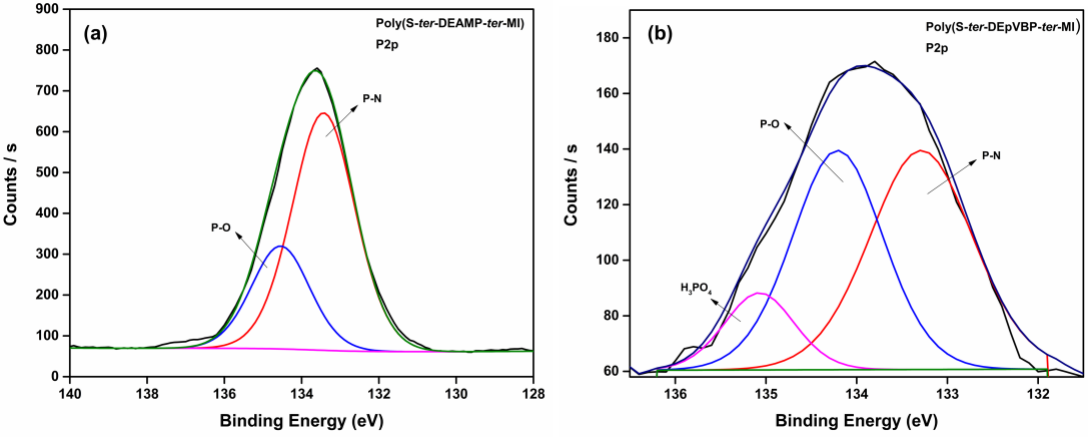
The P2p spectra of chars obtained from poly(S-*ter*-DEAMP-*ter*-MI) (**a**) and poly(S-*ter*-DEpVBP-*ter*-MI) (**b**).

The P2p spectra of chars (Figure 8) were fitted to three peaks located at 133.2, 134.5 and 135.1 eV. The peaks at 133.2 and 134.5 eV can be assigned to the P-N and P-O bonds, respectively [37,38]. The peak at 135.1 eV showed the presence of phosphoric acid [39]. These spectra indicated the formation of P-N- containing compounds in the condensed phase after the thermal degradation of ter-polymers.

**Figure 9:**
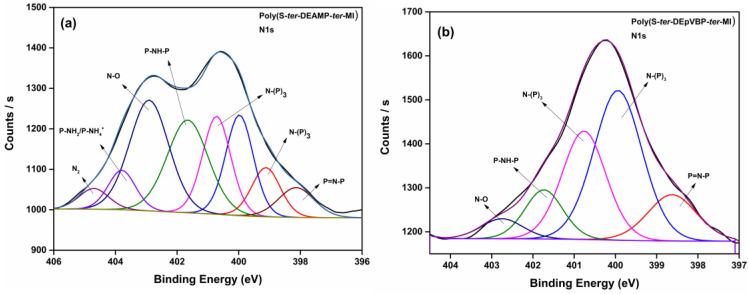
The N1s spectra of chars obtained from poly(S-*ter*-DEAMP-*ter*-MI) (**a**) and poly(S-*ter*-DEpVBP-*ter*-MI) (**b**).

The N1s spectra of the char surfaces produced by ter-polymers poly(S-*ter*-DEAMP-*ter*-MI) and poly(S-*ter*-DEpVBP-*ter*-MI) are presented in Figure 9. Table 5 summarizes the peak fitting data with respect to the bonds and the binding energy. The N1s spectra can be deconvoluted into several peaks that could indicate various chemical environments of nitrogen (N) atoms. As it follows from Table 5, N atoms in the chars from ter-polymers could be linked to P atoms *via* the P=N-P, N-(P)_3_, P-NH-P, or P-NH_2_/P-NH_4_^+^ bonds. However, there was no solid evidence of any possible bonding between N and C atoms: the C-N peaks at 287.9 eV were absent in the C1s spectra. Meanwhile, the N-O bonding was confirmed by both O1s and N1s spectra.

The presence of different types of P-N bonding in char residues of ter-polymers demonstrated that the incorporated P-moieties, DEAMP and DEpVBP, may have interacted with the N-units of MI, forming thermally stable polymeric structures in the condensed phase. The P-N cooperative effects, in our view, may have enhanced the formation of in situ phosphoric acid (*via cis*-elimination), which then underwent a further reaction to produce different P-N intermediates/compounds [42]. It is likely that these P and N interactions improved the phosphorus retention in the condensed phase, promoted charring and stabilization of the residues, thus, leading to a reduced rate of thermal decomposition of poly(S-*ter*-DEAMP-*ter*-MI) and poly(S-*ter*-DEpVBP-*ter*-MI) [8].

**Scheme 2:**
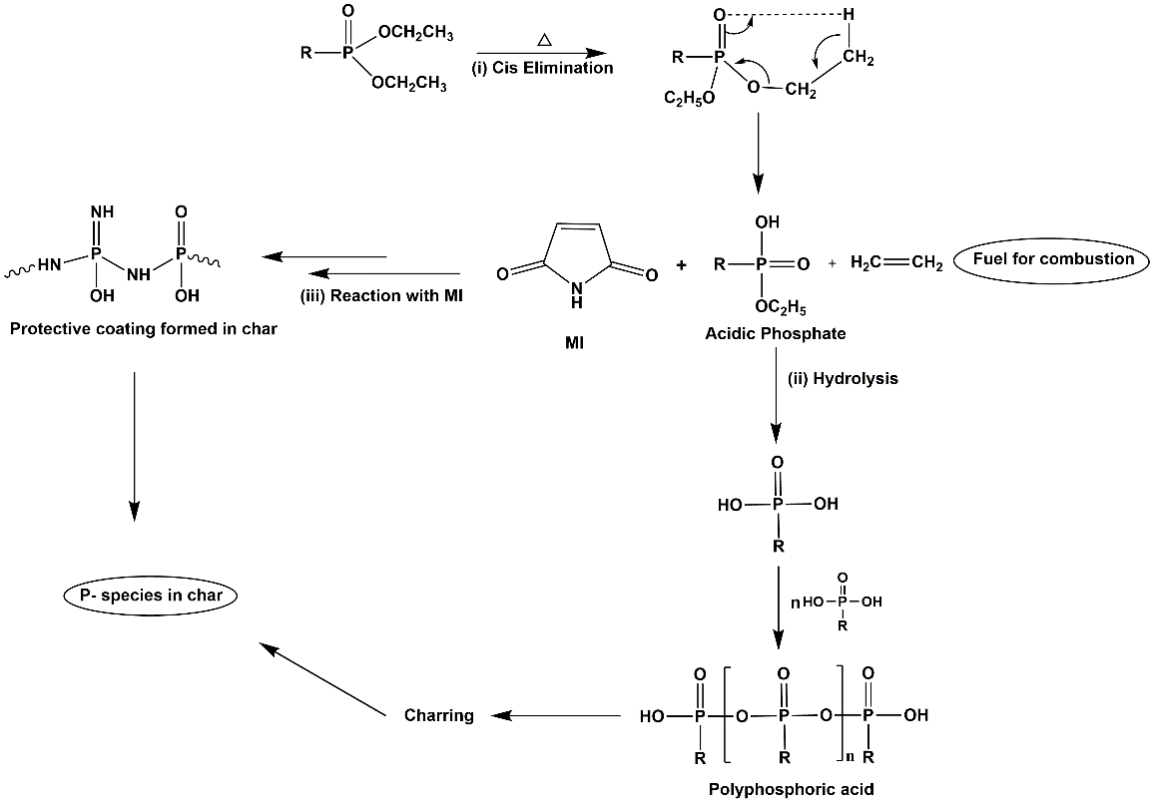
Proposed mechanistic pathways occurring in the condensed phase of ter-polymers (R is a hydrocarbon radical).

Based on the abovementioned results, it is evident that the reactive modifications of PS with P- and N- compounds had altered its thermal degradation pattern and greatly improved the fire resistance of styrenic polymers. The P-containing moieties usually act as fire retardants either in the gaseous- or in the condensed- phase [42]. However, the effect of incorporating P-moieties (DEAMP and DEpVBP) and N-compound (MI) together into the chains of styrenic polymers seemed to result in the synergistic interactions; however, this was noticeable, to a certain degree, in the co-polymer containing P-N comonomer (ADEPMAE). It is also likely that the synergistic effect of phosphorus- and nitrogen-bearing groups promoted an extensive charring process in the condensed phase. Hence, in commensurate with the above observations, the following mechanistic pathways occurring in the condensed phase during thermal degradation of poly(S-*ter*-DEAMP-*ter*-MI) and poly(S-*ter*-DEpVBP-*ter*-MI) can be proposed (Scheme 2).

Initially, at around 200 °C, the phosphorus units in the polymeric chains of ter-polymers underwent a *cis*-elimination, generating acidic phosphates and ethylene, which could act as a fuel (Scheme 2(i)). The acidic phosphates thus formed may undergo a condensation, yielding polyphosphoric acid species (Scheme 2(ii)). The phosphorus acid was also, possibly, retained within the decomposed polymer matrix, promoting charring [43]. Besides, polyphosphoric acids could have covered the surface of the residues, which encourages the formation of compact and phosphorus enriched char layers [43,44]. Meanwhile, the imide group (MI) within the polymeric chains could probably interact with the acidic phosphate to form complex structures containing P-N-P or P=N bonds within a protective coating on the char surfaces (Scheme 2(iii)). These structures can exert the fire retarding effect by isolating the polymer matrix from heat [40,45].

## 3. Materials and Methods

### 3.1. Chemicals and Materials

All the compounds, organic solvents and reagents required for the study were obtained from Merck (former Sigma-Aldrich, Gillingham, UK), apart from ethyl acetate and methanol purchased from Fischer Scientific (Loughborough, UK). Solid reagents were used as received, whilst organic solvents were dried for at least 24 hrs and stored over molecular sieves with pore sizes of 4 Å. 

The syntheses of P- and P-N- containing monomers, diethyl(acryloyloxymethyl)phosphonate (DEAMP), diethyl-*p*-vinylbenzyl phosphonate (DEpVBP), acrylic acid-2-[(diethoxyphosphoryl)methylamino]ethyl ester (ADEPMAE) were carried out according to the procedures used and reported by us previously [15,17,46,47]. The level of purities and chemical structures of DEAMP, DEpVBP and ADEPMAE, or their precursors, were confirmed using the results of gas chromatography-mass spectrometry (GC-MS) (Agilent Technologies, Manchester, UK), ^1^H and ^31^P NMR spectroscopies (Bruker, Coventry, UK), the details of which are already published [13,17]. These monomers (Figure 1, structures I–III) and MI (Figure 1, structure IV) were chemically incorporated into the polymeric chains of polystyrene through a free-radical solution polymerization with styrene. Styrene, needed for each polymerization process, was passed through a prepacked proprietary column to remove small amounts of 4-*tert*-butylcatechol inhibitor from the monomer immediately prior to its use. The preparation of polystyrene (PS) and modified styrenic polymers (co- or ter-polymers) was carried out following the identical synthetic protocol [8] and under the same conditions: using toluene or N,N′-dimethylformamide as a solvent, in the presence of azobisisobutyronitrile (*ca.* 2 g/L) as an initiator, in the inert atmosphere of argon, for 16 hrs, and at 60 ± 0.2 °C. The precipitation, filtration and drying steps of the prepared polymers were also similar as already detailed earlier [8]. For this study, we have prepared four samples of co-polymers, poly(S-*co*-DEAMP), poly(S-*co*-DEpVBP), poly(S-*co*-ADEPMAE), poly(S-*co*-MI), and two samples of ter-polymers, poly(S-*ter*-DEAMP-*ter*-MI) and poly(S-*ter*-DEpVBP-*ter*-MI) to incorporate both P- and N- containing monomeric units. The sample of unmodified PS was used as a control. All the polymers were purified further by reprecipitation and dried in a vacuum oven at 50 ± 1 °C for at least 16 hrs. The preparative data and the structural characterization of the studied polymers were already published elsewhere [8]. In the current paper, the molar percent ratios of the monomeric units in the polymeric chains as well as the contents of P and N (%) were calculated from ^1^H NMR spectra [8] of the synthesized polymers, as given in Table 6. 

### 3.2. Characteriztion Techniques

The Pyrolysis–Gas Chromatography/Mass Spectrometry (Py-GC/MS) studies were performed over a range of temperatures, from 210 °C to 445 °C, on the Pyroprobe 5000 pyrolyzer (CDS Analytical, Inc., Oxford, PA, USA), with a platinum filament, coupled with a gas chromatograph GC7890A (Agilent Technologies, Santa Clara, CA, USA). The separation of pyrolysis products was carried out using a GC column HP-5MS (non-polar, 30 m × 250 μm, 0.25 μm layer thickness, Agilent Technologies, Santa Clara, CA, USA). The used carrier gas was helium with a flowing rate set at 1 mL/min. The chromatograph was equipped with a mass-selective detector (MSD 5975C inert XL E/CI, Agilent Technologies, Santa Clara, CA, USA) with a mass scan range between 15 and 550 amu and the electron impact (EI) at 70 eV. The GC inlet temperature was variable, the oven temperature was programmed as follows: 2 min at 50 °C and heating at 12 °C/min to 280 °C held for 10 min. The polymeric samples were pyrolyzed at temperatures selected from the results of thermogravimetric analysis (TGA), i.e., prior to the significant mass losses (TG and DTG curves recorded at 10 °C/min heating rate in a nitrogen atmosphere are published in [8]). The temperatures of pyrolysis for each polymeric system are given in Table 6. The NIST MS Search 2.0 library was used for identification of pyrolysis gases.

The char analyses were carried out using a number of complementary techniques including solid-state ^31^P Nuclear Magnetic Resonance (NMR) spectroscopy, Scanning Electron Microscopy/Energy Dispersive Spectroscopy (SEM/EDS), Inductively-Coupled Plasma/Optical Emission Spectroscopy (ICP/OES) and X-ray Photoelectron Spectroscopy (XPS). The ^31^P NMR and XPS provided information regarding the chemical environments of P-containing moieties in the char residues. The ICP/OES quantitatively estimated the P-loadings, whereas Energy Dispersive component of SEM/EDS reaffirmed the P-loadings, albeit qualitatively.

The chars for the ^31^P NMR, SEM/EDS and XPS analyses were obtained by taking *ca.* 0.5–1 g of polymers, in a silica crucible, and placing them in a muffle furnace (Carbolite Gero Ltd., Hope, UK). The furnace was heated from 30 °C to 500 °C at a rate of 10 °C/min. The char residues were kept inside the furnace for 20 min and taken out after it cooled down.

The solid-state NMR (^31^P) spectra of char residues obtained from the modified polymers were performed on a 9.4T Bruker Advance II 400 spectrometer (Rheinstetten, Germany) at 162 MHz, with a recycle delay of 120 s. The analysis was carried out with a 4 mm probe head with 1H decoupling (DD). Phosphoric acid was used as an external calibrant. Each spectrum was recorded at MAS with a spinning frequency of 12.5 kHz. All spectra were acquired as a result of 16 scans and were processed using Mnova NMR software.

The Scanning Electron Microscopy (SEM) with Energy Dispersive Spectroscopy (EDS) provision (ThermoScientific, Phenom XL, Melbourne, Australia) was used to obtain the elemental compositions of the char residues, including a control sample from charring of polystyrene, prepared through a solution route in the laboratory at Victoria University. The samples were sputtered with gold (by using a Quorum setup) before mounting them onto the instrument. The images were subjected to the EDS analyses at different points. Given the heterogeneity within each sample, the elemental composition varied according to the point of choice, and hence the quoted values can be only considered as somewhat representative of a particular sample in question. It may be also noted here that the elements that were present in the sample owing to sputtering with Au (including Nb), and other minor ones (particularly F) were eliminated from the final results (therefore, the quoted elemental composition of C, O and P added up to 100%, in each case). Furthermore, it is relevant to note that the technique cannot detect lighter elements such as H, or N.

The X-ray Photoelectron Spectroscopy (XPS) measurements of the residual chars were performed by using an ESCALAB 250Xi spectrometer microprobe (Thermo Fisher Scientific, Oxford, UK) with a focused monochromatic Al Kα X-ray source (hυ = 1486.6 eV, 650 μm spot size) and the photoelectrons were collected using a 180° double-focusing hemispherical analyzer with a dual detector system. Data analysis and fitting were performed with Thermo Avantage software. 

The charring of polymers for Inductively-Coupled Plasma/Optical Emission Spectroscopy (ICP/OES) was carried out by placing into a muffle furnace (ThermoScientific, Thermolyne, Melbourne, Australia) the accurately weighed amounts of each sample inside a porcelain crucible. The temperature of the furnace was slowly ramped from 25 °C to 450 °C (in *ca.* 20 min) and was kept for about *ca.* 15 min. The furnace was then allowed to cool to the ambient temperature, and the crucibles along with their contents were accurately weighed again. The tested samples were first accurately weighed (*ca.* 10-15 mg, in triplicate), and were digested by boiling with a mixture of 5 mL of the AR concentrated HNO_3_. Upon digestion of the contents, the resulting solutions were quantitatively transferred to a 25 mL volumetric flask and the solutions were made up to the mark with deionized water. In the case of incomplete digestion, as inferred from the presence of solid residues, the contents were first filtered using a qualitative filter paper before making up to the required volume. The prepared solutions were transferred to 15 mL sample tubes before being introduced into the ICP/OES instrument (Shimadzu ICPE-9000, Melbourne, Australia). Measurements on each sample were repeated three times, and the corresponding average values calculated. Quantitative assessments of the P content in the samples were deduced by constructing a calibration plot, with a standard solution of KH_2_PO_4_, in accordance with the Beer-Lambert law.

## 4. Conclusions

Generally, polystyrene-based insulation materials are highly flammable/ignitable and require the incorporation of the appropriate fire retardants (FRs) to make them thermally stable for the safe use in the construction sector. Reactive P- and N-containing FRs can alter the thermal degradation patterns and greatly improve the fire resistance of styrenic polymers. In this work, a study of the condensed-phase and gaseous-phase activities of P- and N-bearing FRs chemically bound to the polymeric chains of polystyrene (PS) was carried out for the first time. Deciphering the mechanistic pathways behind the thermal decomposition of the obtained polystyrenes was attempted using a variety of the instrumentation methods. The nature of pyrolysis gases and the associated fragments was evaluated using Pyrolysis-Gas Chromatography/Mass Spectrometry (Py-GC/MS), whilst for the char analyses we employed solid-state ^31^P Nuclear Magnetic Resonance (NMR) spectroscopy, Scanning Electron Microscopy/Energy Dispersive Spectroscopy (SEM/EDS), Inductively-Coupled Plasma/Optical Emission Spectroscopy (ICP/OES) and X-ray Photoelectron Spectroscopy (XPS).

According to the Py-GC/MS results, it can be concluded that reactive modification of PS significantly reduced the evolution of pyrolysis gases including styrene monomer, dimer and trimer, as well as benzaldehyde, α-methylstyrene and acetophenone. These major volatile components are normally responsible for the fast flame spread and the increased toxicity of fires involving styrene-based materials. The gaseous-phase mode of action is likely to involve triethyl phosphate (TEP), detected for poly(S-*co*-DEpVBP), poly(S-*co*-ADEPMAE) and poly(S-*ter*-DEpVBP-*ter*-MI). The formation of TEP, which can give rise to PO⋅ species capable of quenching reactive H⋅ and HO⋅ radicals, is enhanced for poly(S-*co*-ADEPMAE) due to the possible P-N synergism. 

In the case of the prepared ter-polymers, a suppressed release of pyrolysis gases and related fragments to the gaseous phase may be caused by charring processes, which suggest that the condensed-phase mode of action prevails over the gaseous-phase activity. This effect was more notable for poly(S-*ter*-DEAMP-*ter*-MI), which could be explained by the formation of a compact and resilient protective char layer. Moreover, the results of EDS, ICP/OES and XPS indicated that the phosphorus species were retained in the structures and on the surfaces of chars, which was also evident from the solid-state ^31^P NMR spectra. The ‘phosphorus’ acid species generated during the thermal cracking of the phosphonate groups possibly resulted in phosphorylation of aromatic rings of the modified polystyrenes, subsequently forming char precursors and cross-linking polymeric chains, and ultimately improving the condensed-phase activity of FRs. The incorporation of P-moieties, DEAMP and DEpVBP, and N-compound, MI, together into the chains of styrenic polymers promoted the extensive charring processes in the condensed phase owing to the synergistic P and N interactions. 

## Figures and Tables

**Table 1:** The assignment of chromatographic peaks and mass spectral signals for the gaseous products formed as a result of PS decomposition *.

Compound Name	*m*/*z*	Chemical Structure	RT (min) at 260 °C	RT (min) at 415 °C
Toluene	92	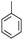	3.44	3.44
Ethylbenzene	106	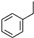	-	4.71
Styrene-monomer	104	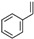	5.17	5.15
Benzaldehyde	106	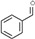	6.17	6.16
α-Methylstyrene	106	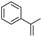	-	6.48
Limonene	136	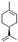	-	7.15
Phenylacetaldehyde	120	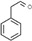	7.37	7.35
Acetophenone	120	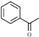	7.68	7.68
Dihydroxyacetophenone	152	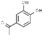	7.76	-
Bibenzyl	182	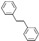	-	13.13
Diphenylpropane	196	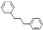	-	14.42
Stilbene	180	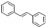	-	14.96
Styrene-dimer	208	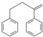	-	15.06
Styrene-trimer	314	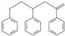	-	20.77

* The GC/MS was used to elucidate the composition of the evolved gases. A quantitative GC analysis of the gas composition will be possible in the future, after a calibration with the identified volatile compounds is carried out, which was not the purpose of the current study. However, GC/MS option provided a realistic estimation with respect to major and minor components of the evolved gases.

**Table 2:** The proposed assignment of some mass spectral signals to the gaseous fragments formed by the decomposition of co-polymers.

Poly(S-*co*-DEAMP)		Poly(S-*co*-ADEPMAE)		Poly(S-*co*-MI)	
Ion Structure	*m*/*z*	Ion Structure	*m*/*z*	Ion Structure	*m*/*z*
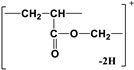	83	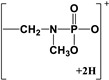	124	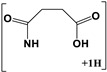	115
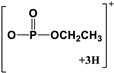	111	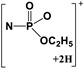	124	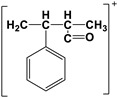	158
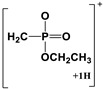	123	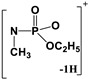	136	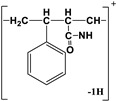	172
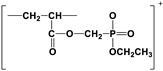	193	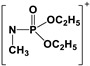	166	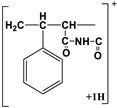	189

**Table 3:** The char yields, phosphorus contents and percentage retention of phosphorus in the condensed phase.

Polymeric Sample	P Content in Polymer (wt. %)	Char Yield (wt. %)	P Content in Char (wt. %) *	Theoretical P_max_ Loading in Char (wt. %) ^#^	Actual P Retention in Char (wt. %) ^$^	P Content in Char from EDS (wt. %)
Poly(S-*co*-DEAMP)	3.07	21.37	9.73	14.4	67.6	-
Poly(S-*co*-DEpVBP)	1.87	21.01	3.50	8.90	39.3	-
Poly(S-*co*-ADEPMAE)	2.55	14.29	6.10	17.8	34.5	4.5
Poly(S-*ter*-DEAMP-*ter*-MI)	2.95	33.00	3.47	8.94	38.8	7.2
Poly(S-*ter*-DEpVBP-*ter*-MI)	1.59	34.83	2.20	4.57	48.1	5.1

* Average of triplicate runs. ^#^ P loading (theoretical maximum) in the char = [(P content in polymer)/(char yield)] × 100. ^$^ Actual P retention in the char = [(P content in char)/(theoretical P_max_ loading)] × 100. For example, for poly (S-*co*-DEAMP): Theoretical P_max_ loading = [3.07/21.37] × 100 = 14.4 wt. % (assuming 100% P retention); Actual P retention in char = [9.73/14.4] × 100 = 67.6 wt. %.

**Table 4:** The peak assignment of C1s and O1s spectra of chars.

Bonds	Assignment	Binding Energy (eV)	References
**C1s**			
C-C/C=C	aliphatic/aromatic or sp^2^ bonded carbons	284.7–284.8	[33,34,35,36]
C-O	ether or hydroxyl bonded carbon	285.9–286.0	
C=O	carbonyl groups	287.1	
COO	carboxyl or ester groups	288.1	
**O1s**			
O=C	carbonyl group	531.1	[33,35,36]
O-C	bonded to aliphatic	532.2–532.6	
O-C	bonded to aromatic	533.2–533.7	
O=N	bonded to nitrogen	534.1–534.7	
H-O-H	absorbed water	535.2–535.5	

**Table 5:** The peak data for N1s spectra for chars obtained from ter-polymers.

Bonds	Binding Energy (eV)		References
	Poly(S-*ter*-DEAMP-*ter*-MI)	Poly(S-*ter*-DEpVBP-*ter*-MI)	
P=N-P	398.1	398.6	[39,40,41]
N-(P)_3_	399.9–400.7	399.9–400.7	
P-NH-P	401.6	401.7	
N-O (oxidized N)	402.9	402.7	
P-NH_2_/P-NH_4_^+^	403.7	-	
N_2_	404.7	-	

**Table 6:** Characterization of studied polymers.

Polymer Sample	P Content wt. %	N Content wt. %	[S]:[M_1_]:[M_2_] * in Polymer, mol %	T_pyrolysis,_ °C
**PS**	-	-	100:0.00:0.00	260, 415
**Poly(S-*co*-DEAMP)**	3.07	-	88.3:11.7:0.00	210, 325, 380
**Poly(S-*co*-DEpVBP)**	1.87	-	93.1:6.90:0.00	240, 355, 445
**Poly(S-*co*-ADEPMAE)**	2.55	1.15	90.1:9.90:0.00	250, 300, 400
**Poly(S-*co*-MI)**	-	3.72	72.8:0.00:27.2	210, 420
**Poly(S-*ter*-DEAMP-*ter*-MI)**	2.95	3.07	63.7:11.0:25.3	240, 320, 375
**Poly(S-*ter*-DEpVBP-*ter*-MI)**	1.59	3.63	65.6:5.70:28.7	270, 375

* [M_1_] is P- or P-N- monomer (i.e., DEAMP/DEpVBP/ADEPMAE), [M_2_] is N- monomer (MI).

## Data Availability

All data are already presented in detail in the manuscript in each section under the corresponding title and in the appendices.

## Supplementary Materials

The following supplementary information can be downloaded at: https://www.mdpi.com/article/10.3390/molecules28010278/s1, Figure S1: Gas chromatograms of pyrolysis gases released upon the decomposition of PS at 260 °C (black) and 415 °C (blue); Figure S2: Mass spectrum of gases evolved as a result of PS pyrolysis at 415 °C (20.769 min); Figure S3: Mass spectrum of gases formed as a result of poly(S-*co*-DEpVBP) pyrolysis at 355 °C (15.76 min); Figure S4: Mass spectrum of gases formed as a result of poly(S-*co*-DEpVBP) pyrolysis at 445 °C (21.49 min); Figure S5: Gas chromatograms of pyrolysis gases released upon the decomposition of poly(S-*co*-MI) at 210 °C (black) and 420 °C (blue); Figure S6: Mass spectrum of gases formed as a result of poly(S-*co*-MI) pyrolysis at 420 °C (15.698 min); Figure S7: Gas chromatograms of pyrolysis gases evolved during the decomposition of poly(S-*ter*-DEAMP-*ter*-MI) at 240 °C (black), 320 °C (blue) and 375 °C (red); Figure S8: Mass spectrum of gases evolved as a result of poly(S-*ter*-DEAMP-*ter*-MI) pyrolysis at 375 °C (20.763 min); Figure S9: Gas chromatograms of pyrolysis gases evolved during the decomposition of poly(S-*ter*-DEpVBP-*ter*-MI) at 270 °C (black) and 375 °C (blue); Figure S10: Mass spectrum of gases evolved as a result of poly(S-*ter*-DEpVBP-*ter*-MI) pyrolysis at 375 °C (20.763 min); Figure S11: The solid-state ^31^P NMR spectra of char residues obtained from the samples of poly(S-*co*-ADEPMAE) (a), poly(S-*ter*-DEAMP-*ter*-MI) (b), poly(S-*ter*-DEpVBP-*ter*-MI) (c).

## Sample Availability

Samples of the compounds are not available from the authors.
