# Supplementary material for: Gaseous- and Condensed-Phase Activities of Some Reactive P- and N-Containing Fire Retardants in Polystyrenes
Source: Molecules. 2022 Dec 29;28(1):278. doi: 10.3390/molecules28010278 (PMC9822389; doi:10.3390/molecules28010278)
Supplement: Supplementary file 1 [file molecules-28-00278-s001.zip › molecules-2101224-supplementary.pdf]

Supplementary Information (SI)

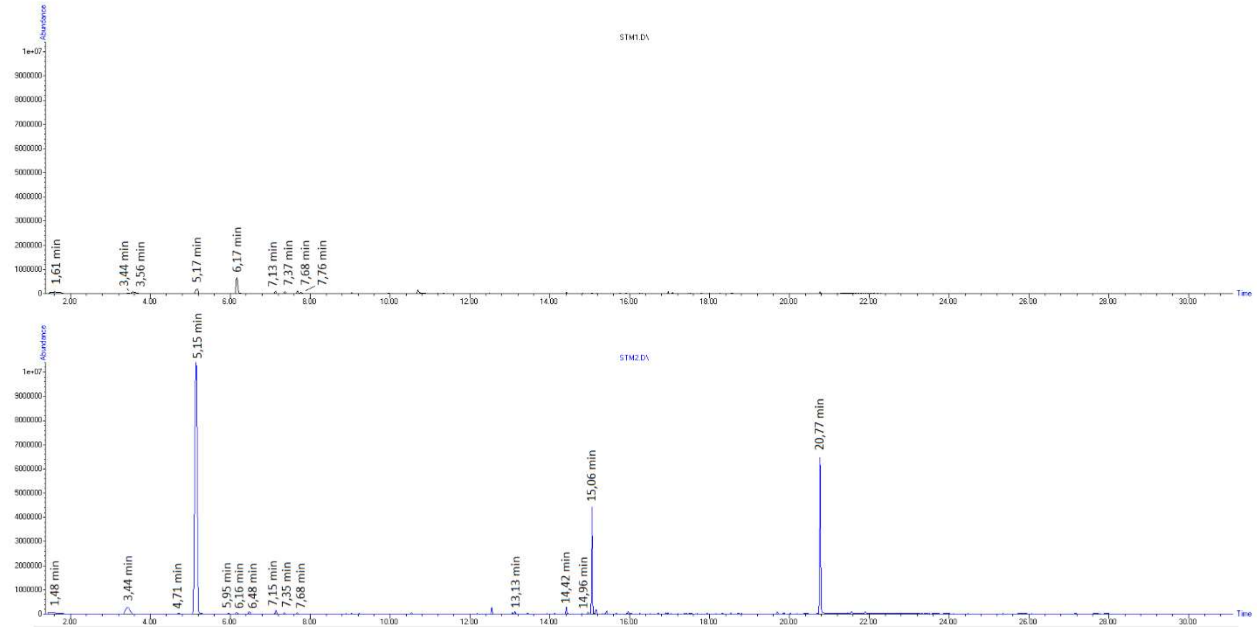

Figure S1. Gas chromatograms of pyrolysis gases released upon the decomposition of PS at 260 °C (black) and 415 °C (blue).

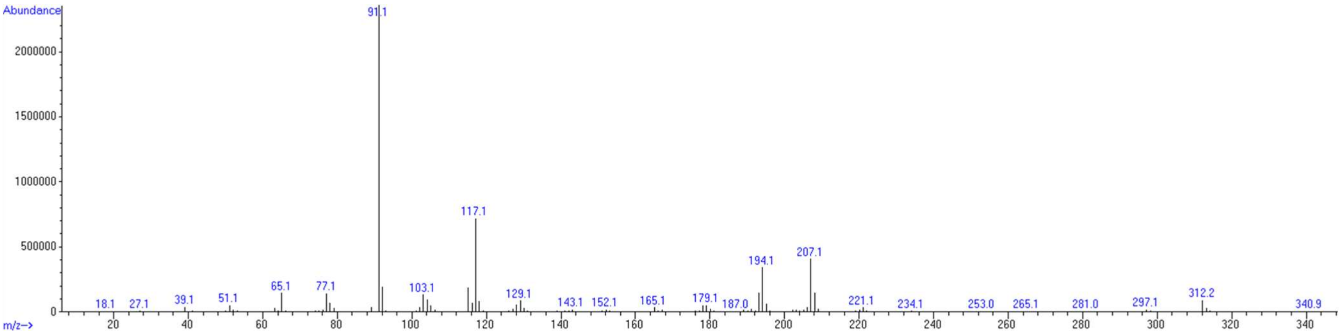

Figure S2. Mass spectrum of gases evolved as a result of PS pyrolysis at 415 °C (20.769 min).

Figure S3. Mass spectrum of gases formed as a result of poly(S-*co*-DEpVBP) pyrolysis at 355 ^{o}C (15.76 min).

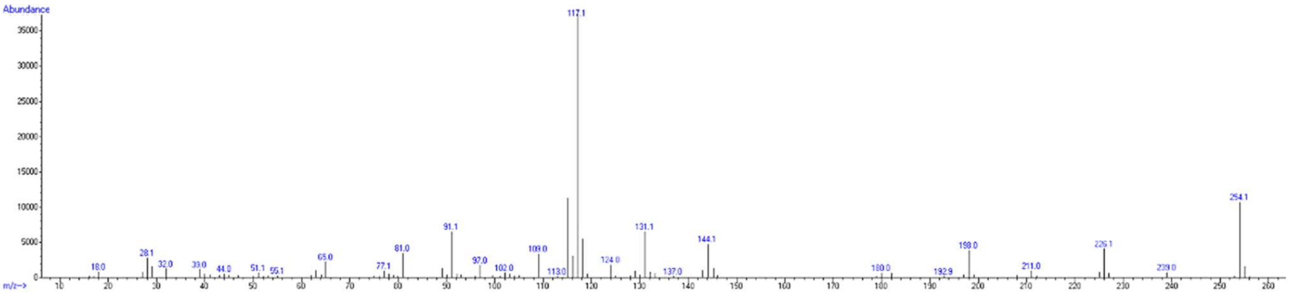

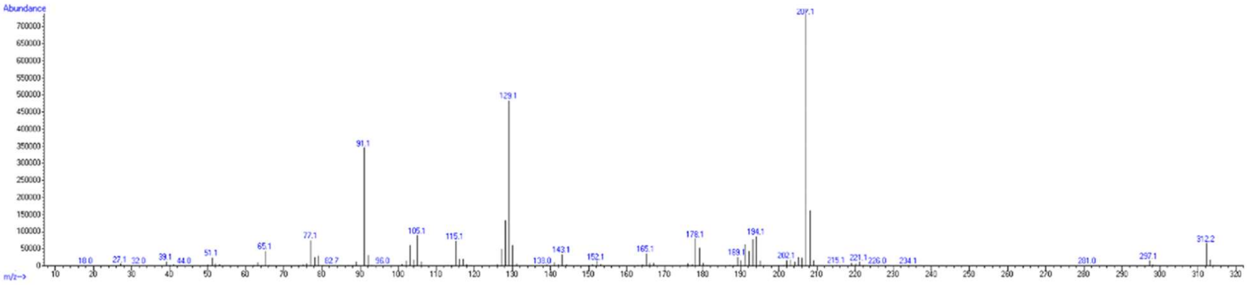

Figure S4. Mass spectrum of gases formed as a result of poly(S-*co*-DEpVBP) pyrolysis at 445 ^{o}C (21.49 min).

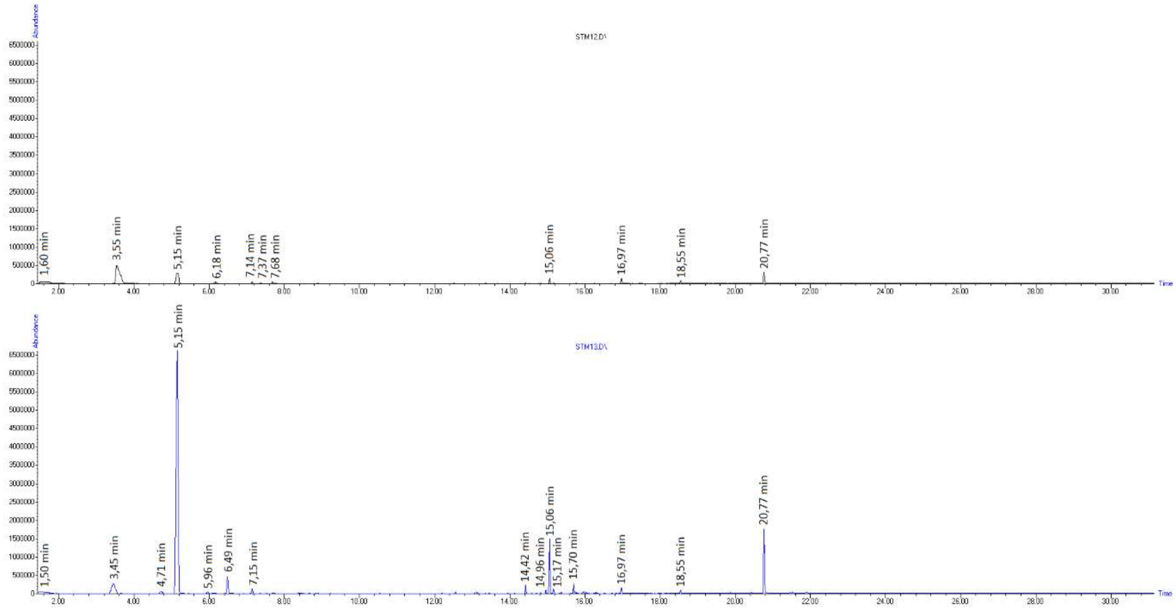

Figure S5. Gas chromatograms of pyrolysis gases released upon the decomposition of poly(S-*co*-MI) at 210 °C (black) and 420 °C (blue).

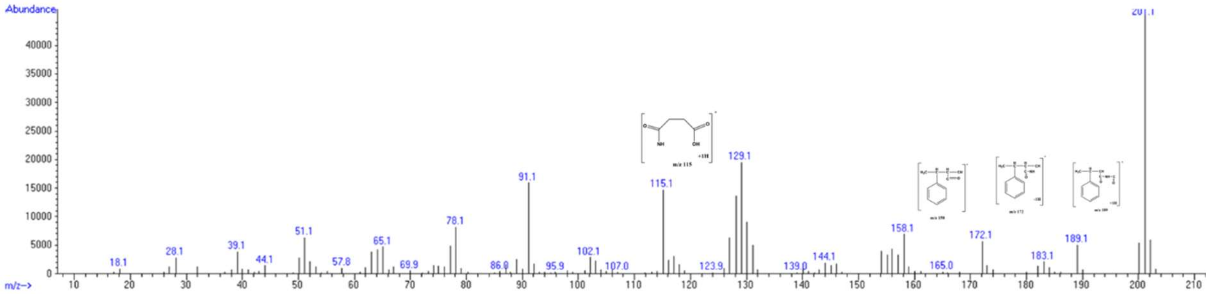

Figure S6. Mass spectrum of gases formed as a result of poly(S-*co*-MI) pyrolysis at 420 °C (15.698 min).

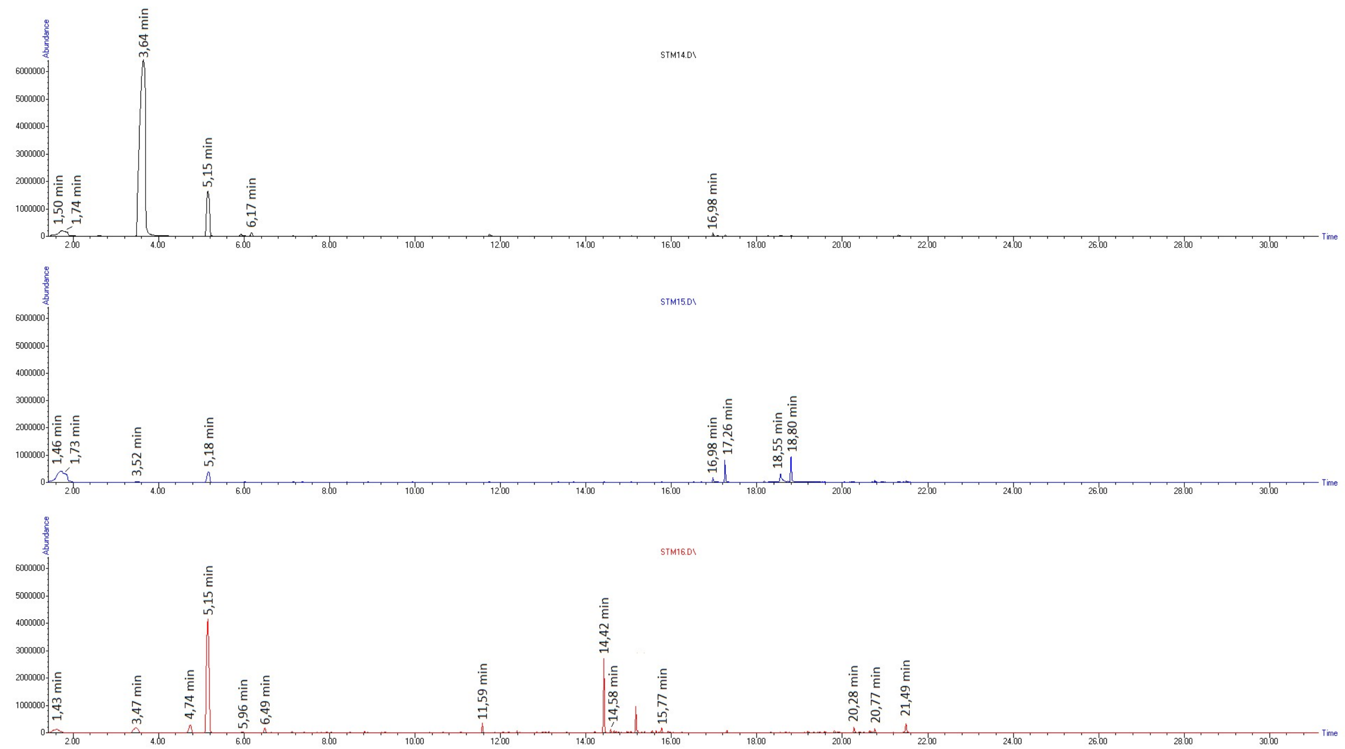

Figure S7. Gas chromatograms of pyrolysis gases evolved during the decomposition of poly(S-*ter*-DEAMP-*ter*-MI) at 240 °C (black), 320 °C (blue) and 375 °C (red).

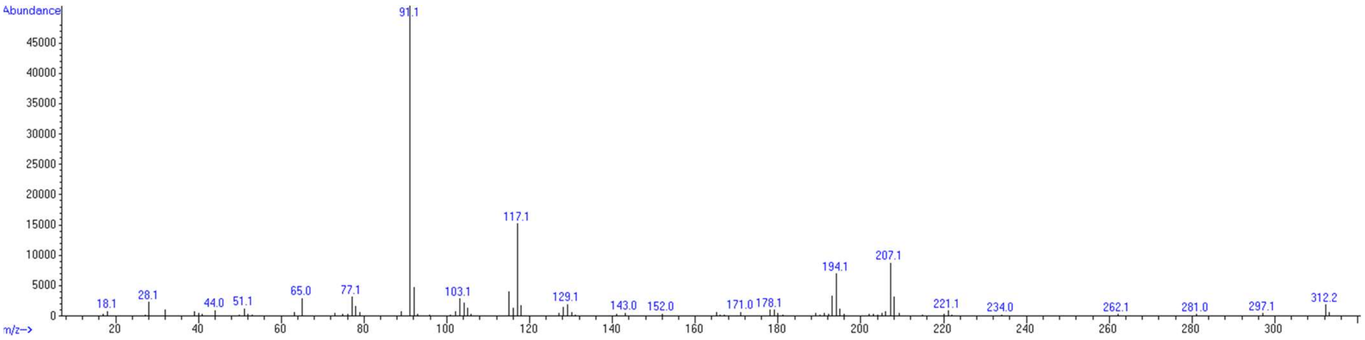

Figure S8. Mass spectrum of gases evolved as a result of poly(S-*ter*-DEAMP-*ter*-MI) pyrolysis at 375 °C (20.763 min).

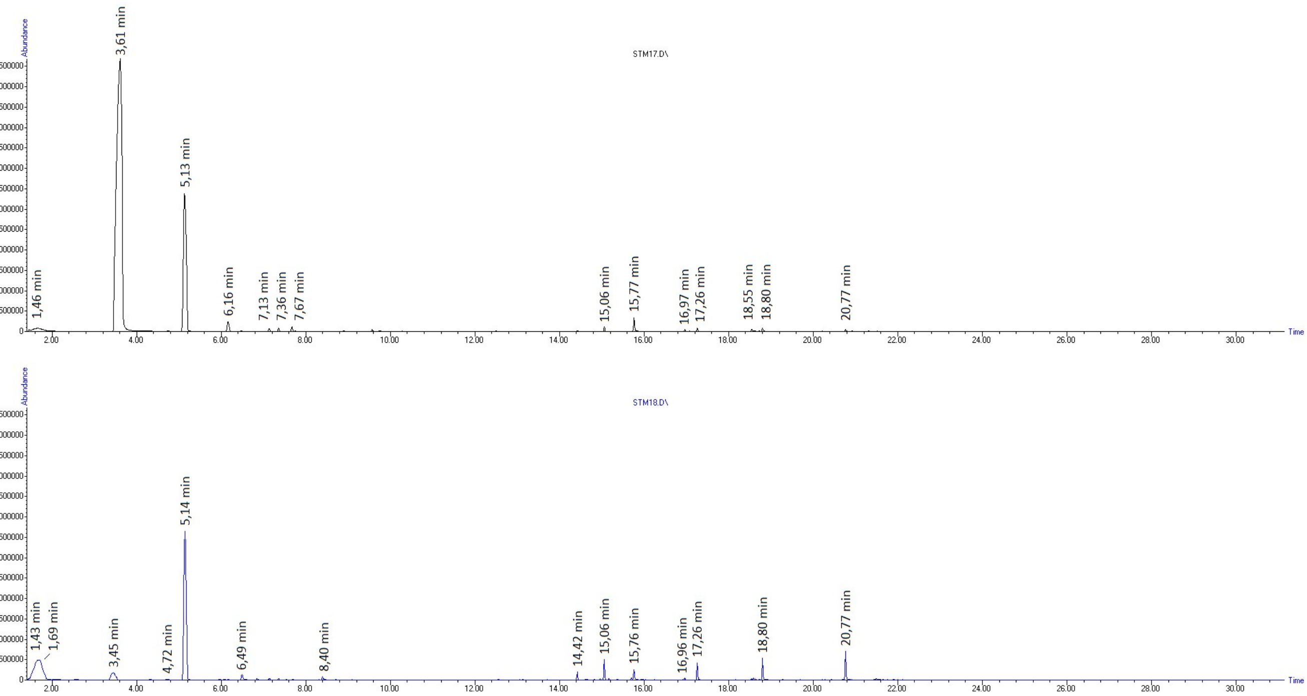

Figure S9. Gas chromatograms of pyrolysis gases evolved during the decomposition of poly(S-*ter*-DEpVBP-*ter*-MI) at 270 ^{o}C (black) and 375 ^{o}C (blue).

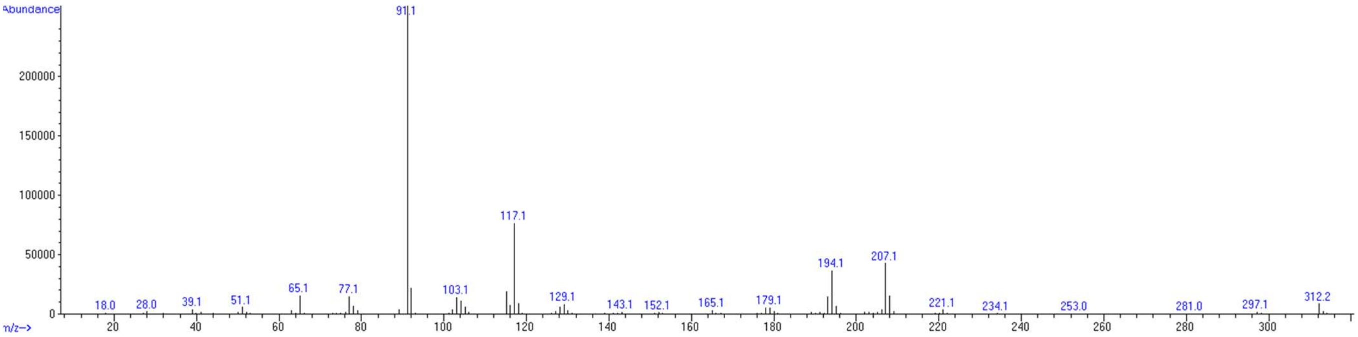

Figure S10. Mass spectrum of gases evolved as a result of poly(S-*ter*-DEpVBP-*ter*-MI) pyrolysis at 375 ^{o}C (20.763 min).

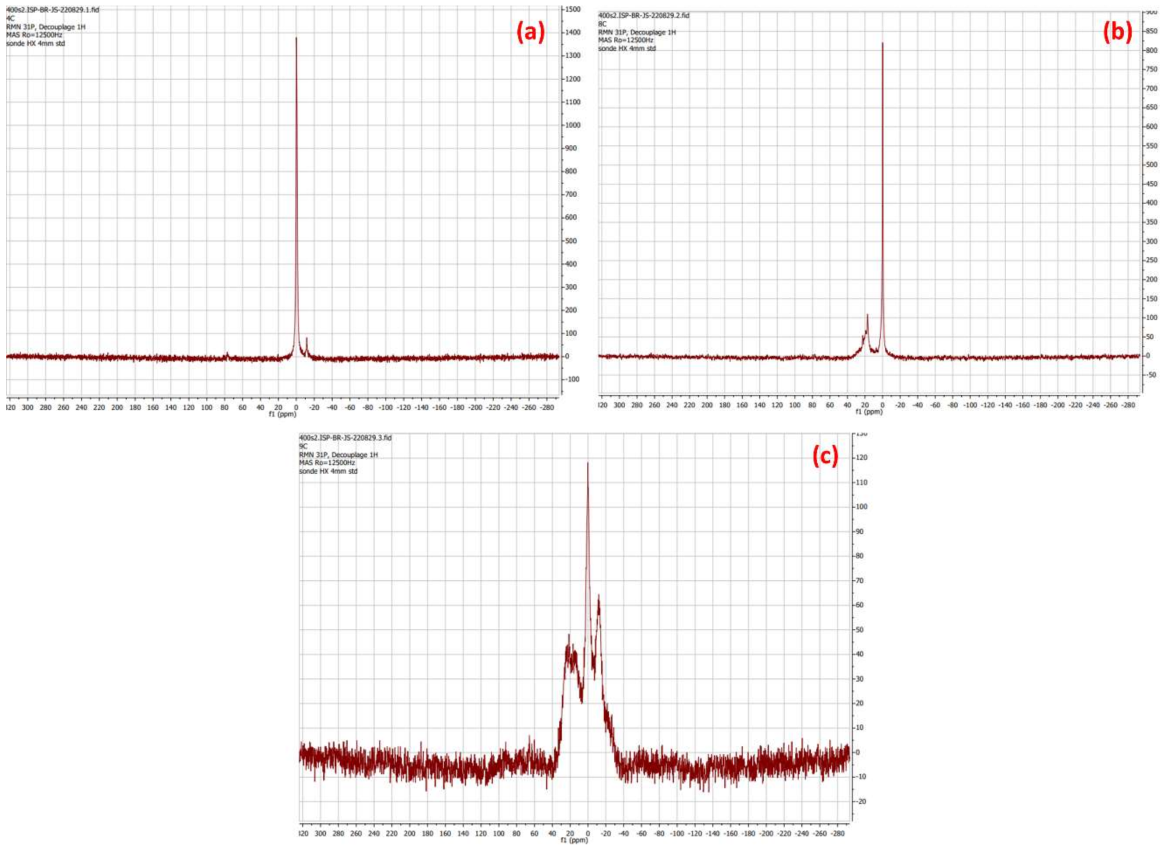

Figure S11. The solid-state ^{31}P NMR spectra of char residues obtained from the samples of poly(S-*co*-ADEPMAE) (a), poly(S-*ter*-DEAMP-*ter*-MI) (b), poly(S-*ter*-DEpVBP-*ter*-MI) (c).
